# A generalised approach to the study and understanding of adaptive evolution

**DOI:** 10.1111/brv.12910

**Published:** 2022-10-12

**Authors:** Pim Edelaar, Jun Otsuka, Victor J. Luque

**Affiliations:** ^1^ Department of Molecular Biology and Biochemical Engineering Universidad Pablo de Olavide Carretera Utrera km.1 41013 Seville Spain; ^2^ Swedish Collegium for Advanced Study Thunbergsvägen 2 SE‐75238 Uppsala Sweden; ^3^ Department of Philosophy Kyoto University Yoshida‐Hommachi, Sakyo Kyoto 606‐8501 Japan; ^4^ RIKEN Center for Advanced Intelligence Project 1‐4‐1 Nihonbashi Tokyo 103‐0027 Japan; ^5^ Department of Philosophy University of Valencia Av. de Blasco Ibáñez, 30 46010 València Spain

**Keywords:** evolutionary theory, adaptation, non‐genetic inheritance, plasticity, causal modelling, Price equation, evolutionary synthesis

## Abstract

Evolutionary theory has made large impacts on our understanding and management of the world, in part because it has been able to incorporate new data and new insights successfully. Nonetheless, there is currently a tension between certain biological phenomena and mainstream evolutionary theory. For example, how does the inheritance of molecular epigenetic changes fit into mainstream evolutionary theory? Is niche construction an evolutionary process? Is local adaptation *via* habitat choice also adaptive evolution? These examples suggest there is scope (and perhaps even a need) to broaden our views on evolution. We identify three aspects whose incorporation into a single framework would enable a more generalised approach to the understanding and study of adaptive evolution: (*i*) a broadened view of extended phenotypes; (*ii*) that traits can respond to each other; and (*iii*) that inheritance can be non‐genetic. We use causal modelling to integrate these three aspects with established views on the variables and mechanisms that drive and allow for adaptive evolution. Our causal model identifies natural selection and non‐genetic inheritance of adaptive parental responses as two complementary yet distinct and independent drivers of adaptive evolution. Both drivers are compatible with the Price equation; specifically, non‐genetic inheritance of parental responses is captured by an often‐neglected component of the Price equation. Our causal model is general and simplified, but can be adjusted flexibly in terms of variables and causal connections, depending on the research question and/or biological system. By revisiting the three examples given above, we show how to use it as a heuristic tool to clarify conceptual issues and to help design empirical research. In contrast to a gene‐centric view defining evolution only in terms of genetic change, our generalised approach allows us to see evolution as a change in the whole causal structure, consisting not just of genetic but also of phenotypic and environmental variables.

## I. INTRODUCTION

Evolutionary biology has made large impacts on our understanding of the world, and on the management of pest organisms, diseases, agriculturally important organisms, threatened species, etc. Initially grounded on basic observations and inferences (Darwin, 1859), it has since been combined and expanded successfully with new data and insights, most notably the discovery of genes and DNA, the importance of neutral (stochastic) processes, and the development of quantitative genetics. However, despite this success and flexibility, it has been receiving an increasing amount of criticism (Endler & McLellan, 1988; Pennisi, 2008; Laland *et al*., 2014; Welch, 2017; Huneman & Walsh, 2017; Jablonka, 2017; Svensson, 2022). This discussion is in part fuelled by discoveries in ecology, cell biology, developmental biology and genomics, but also due to some longer‐standing unresolved issues (Endler & McLellan, 1988; Pigliucci & Müller, 2010; Tanghe *et al*., 2018; Lewens, 2019; Baedke, Fabregas‐Tejeda & Vergara‐Silva, 2020; Svensson, 2022). To introduce this discussion, below we review three examples of biological phenomena which appear not to fit comfortably into mainstream evolutionary theory. [It is hard to define ‘mainstream evolutionary theory’, but it is used herein based on our appreciation of current usage in the literature (including textbooks) by the majority of people. This does not mean we neglect ongoing modern developments, but we feel these are not yet mainstream]. Our view is that additional combination and expansion of evolutionary theory with new insights might be necessary. In the remainder of this paper we derive and suggest a means to do this, and discuss the implications of this approach.

### (1) Examples of tension between certain biological phenomena and mainstream evolutionary theory

#### (a) How does the inheritance of molecular epigenetic changes fit into mainstream evolutionary theory?

Originally thought to be part only of gene regulation during ontogeny, a number of molecular epigenetic changes are now known to be transmitted from parents to offspring generations. Some of these transmitted epigenetic changes causing phenotypic effects in parents and offspring are beneficial responses to environmental stressors and cues, so are involved in adaptive phenotypic plasticity. An illuminating example is the study by Dias & Ressler (2014), who conditioned F0 male mice to fear an odour by pairing presentation to the odour with mild electric shocks prior to breeding. They then tested response to the odour in F1 and F2 offspring which were never exposed to the electrical shocks, yet also showed a heightened startle response to this odour. This response was not socially transmitted from the F0 generation, since the behavioural sensitivity to the odour and the associated neuroanatomical changes were also observed in F1 and F2 offspring produced *via in‐vitro* fertilization and cross‐fostering. Bisulfite sequencing of sperm DNA from conditioned F0 mice and their F1 offspring revealed the presence of 5′–C–phosphate–G–3′ (CpG) hypomethylation in an odour receptor gene, providing the likely mechanism for the intergenerational transmission of an adaptively acquired fear. For other examples and the variety of molecular epigenetic changes involved and their transmission, see Wang, Liu & Sun (2017), Danchin *et al*. (2019*b*), Adrian‐Kalchhauser *et al*. (2020) and references therein. All these processes create parent–offspring similarity, or more generally speaking they create non‐genetic channels for passing on information between generations (Griffiths, 2017). This raises some serious questions. Can adaptive traits that have been acquired by parents in response to environmental challenges be transmitted to offspring? This question has been largely sidelined since Weismann (1883) showed experimentally that changes to the soma were not passed on to the germ line. So, is the common assumption in (quantitative) genetics that heritability reflects the genetic basis of traits an excessive simplification? Quoting Adrian‐Kalchhauser *et al*. (2020, p. 1078): ‘For evolutionary biologists, the phenotypic impact of inherited “non‐genetic” factors and their potential contribution to adaptation and diversification are pressing issues […] Must we fundamentally revise our understanding of inheritance to incorporate these new insights?’

#### (b) Is niche construction an evolutionary process?

Niche construction refers to the modification of selective environments by organisms (Laland, Matthews & Feldman, 2016). In its broadest formulation, this modification is not necessarily adaptive, and also may affect the selective environment of non‐focal (including heterospecific) organisms. The consequences of organismal activity on the selective environment have been part of evolutionary theory at least since Darwin's writings on earthworms and corals (Darwin, 1842, 1881), and featured in the earliest mathematical models of natural selection (Fisher, 1930; Haldane, 1932). Well‐known general examples are fitness that depends on allele or genotype frequency, sexual selection (preference in the choosy sex determines fitness variation in the other sex), and predator–prey interactions. In all these cases the organism influences its own evolution by modifying the selective conditions, by being both the object of natural selection and the creator and modifier of the conditions of that selection (Levins & Lewontin, 1985; Odling‐Smee, Laland & Feldman, 2003; Laland, Odling‐Smee & Feldman, 2019). Following that observation, it has been claimed that ‘When such modifications alter natural selection pressures, evolution *by* [our italics] niche construction is a possible outcome’ (Laland *et al*., 2016, p. 192), and even that ‘Niche construction should be regarded, after natural selection, as a second major participant in evolution’ (Odling‐Smee *et al*., 2003, p. 2). In response, others (Scott‐Phillips *et al*., 2014, pp. 1234–1235) have stated that ‘this claim conflates evolutionary processes with the *causes* [our italics] of those processes. Niche construction, like all environmental change, can cause evolutionary processes to occur, but this does not make it an evolutionary process itself […] Of the different evolutionary processes (e.g. natural selection, genetic drift, mutation, and migration) only natural selection can explain adaptation. We do not see how niche construction either generates or sorts genetic variation independently of these other processes, or how it changes gene frequencies in any other way’. Advocates of niche construction theory, by contrast, conceive of evolutionary processes more broadly, as anything that systematically biases the direction or rate of evolution, in which case niche construction would be an evolutionary process (Uller & Helanterä, 2017; Laland *et al*., 2019). This evolutionary role of niche construction is hotly debated (e.g. Wray *et al*., 2014; Laland *et al*., 2014; Gupta *et al*., 2017*a*,*b*; Feldman, Odling‐Smee & Laland, 2017; Uller & Helanterä, 2017; Constant *et al*., 2018; Fokkema *et al*., 2021).

#### (c) Is local adaptation via habitat choice also adaptive evolution?

Habitat choice is very common in nature, both at the interspecific level (different species prefer different habitats) and at the intraspecific level (different individuals prefer different habitats; Jaenike & Holt, 1991). Given that environments are heterogeneous and that this may result in a lot of variation in expected fitness, it is not surprising that habitat choice has evolved as a means to increase fitness (Rausher, 1984). It has long been known that individual habitat choice can promote adaptation in heterogeneous environments (Levins, 1963). Whereas random dispersal between populations (i.e. intraspecific habitat choice is absent) tends to homogenise populations and thereby decreases local adaptation, when individuals can influence their movement in order to end up in environments that are favourable to them given their phenotype, local adaptation can actually be maintained or even enhanced by dispersal (Jacob *et al*., 2017). In some circumstances (e.g. within‐habitat mating) it may lead to assortative mating (Diehl & Bush, 1989; Edelaar *et al*., 2019) and even the formation of locally adapted, ecologically distinct species (Maynard Smith, 1966; Rice & Salt, 1988).

However, that leaves us with a conundrum (Edelaar, Siepielski & Clobert, 2008; Edelaar & Bolnick, 2012; Bolnick & Otto, 2013): is (presumably) genetic structuring into locally adapted populations and even new species an example of adaptive evolution, given that it is not driven by currently acting natural selection? Of course natural selection was involved in the prior evolution of habitat choice, but the actual divergence is not driven by current natural selection if there is no systematic differential reproduction among phenotypes. Some would argue that instead it is driven by individuals selecting their habitats in order to *avoid* natural selection. If adaptive population divergence by habitat choice is not adaptive evolution (even though it has the same outcome as divergent natural selection), then why not? And if it is, then what is the driver of adaptive evolution, if natural selection did not operate during the divergence? And which trait is it then that presumably evolves?

### (2) Objective

To address these and other phenomena and conceptual issues that appear to fit uncomfortably in evolutionary theory as currently used, we present a generalised approach to the study of adaptive evolution. For reasons explained below, this approach is generalised in the sense that we include (*i*) a broadened view of extended phenotypes, (*ii*) that traits can respond to each other, and (*iii*) that inheritance can be non‐genetic. None of these elements are new by themselves (in fact they each have a large literature addressing them), and neither is the approach we take. The novelty and value lie in integrating into a single model what so far have been relatively separate treatments, and the insights that come from that. The model we develop can act as a general framework to address and discuss evolutionary questions of a practical/empirical nature but especially of a conceptual nature. After presenting the model, we therefore revisit the three problematic biological phenomena outlined in Section I.1 and reinterpret them in view of our framework. By doing so, we aim to show how the framework can act as a heuristic tool that helps to pose and answer questions (including empirical ones), and can potentially resolve or at least clarify some older and recent discussions in the literature regarding the structure and future of evolutionary theory. Some of our views and interpretations might not be the same as those of the reader, and some inferences might seem strange or even be uncomfortable, but we hope the reader is willing to assess the logic and value of our messages after our arguments have been fully laid out.

## II. MODELLING GENERALISED ADAPTIVE EVOLUTION

### (1) Causal modelling as a tool for insight and prediction

Our heuristic model is based on causal modelling. Causal modelling can serve to improve our understanding of evolution either conceptually (including in practical investigations, e.g. by examining how and whether hypothetical mechanisms may lead to evolutionary change) or empirically (by actually estimating or confirming causal hypotheses based on data). Causal modelling visually represents a causal hypothesis about which variables affect each other with a directed graph, and examines how it generates probability distributions over the variables. Inversely, it infers the underlying causal structure from observed statistical data. Its history dates back to Wright's path analysis (Wright, 1934; Spirtes, Glymour & Scheines, 1993; Pearl, 2000), and it is being applied more and more frequently in the biological literature (e.g. Arnold, 1983; Bookstein & Crespi, 1989; Kingsolver & Schemske, 1991; Queller, 1992; Otto, Christiansen & Feldman, 1995; Conner, 1996; Frank, 1997; Shipley, 2000; Scheiner, Mitchell & Callahan, 2000; Rice, 2004; Latta & McCain, 2009; Morrissey, 2014; Otsuka, 2015, 2016; Okasha, 2016; Araya‐Ajoy, Westneat & Wright, 2020; Henshaw, Morrissey & Jones, 2020; Queller, 2020). Our motivation in this paper is conceptual and heuristic, with our focus being on determining causal conditions which lead to adaptive evolution, and not on empirically finding and estimating such causal relationships. Readers interested in empirical issues of causal discovery and estimation are referred to Valente *et al*. (2010), Morrissey (2014), and Otsuka (2016).

**Fig. 1:**
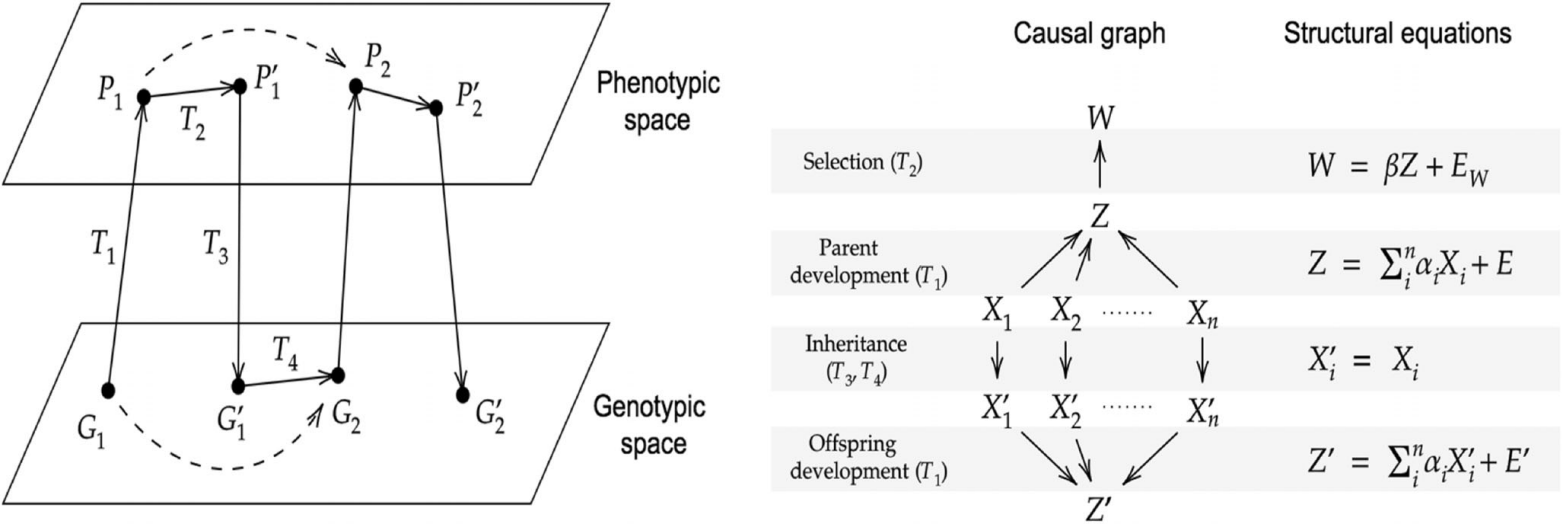
Lewontin's (1974) scheme of evolutionary processes in phenotypic/genotypic spaces (left). The four transition steps (*T*_1_–*T*_4_) represent the causal processes that an evolving population undergoes. These causal processes are modelled with a causal graph over relevant variables (right), and each causal connection is quantified by structural equations. *Z* and *X* represent the individual phenotypic and genotypic values for the population quantities *P* and *G*, respectively. *β* is the selection gradient, *α* is an additive genetic effect, and *E* is an independent noise term. (Note that in the panel on the left the prime indicates different time steps within a generation and different generations are indicated with a numerical subscript for *P* and *G*; in the panel on the right the different generations are indicated with a prime, and the numerical subscripts refer to different genes). Combined with the Price equation (see Section IV), the model then induces a formula (in this case, the breeder's equation) that gives the evolutionary change in phenotype between two generations (the dotted line from *P*_1_ to *P*_2_). Adapted from Otsuka (2019*b*).

Evolution can be conceptualised as trajectories in phenotypic as well as genotypic spaces mediated by four major transition steps, namely: (*i*) development from zygotes into adult form; (*ii*) change in phenotypic distribution due to natural selection or other evolutionary processes; (*iii*) gamete production; and (*iv*) formation of new zygotes (Lewontin, 1974; Fig. 1 left). The causal analysis of an evolutionary process proceeds by modelling these four steps in terms of causal graphs, and then specifying the functional form of each causal connection. From a fully parameterised model one can calculate a probability distribution over the variables. Combined with the Price equation discussed in Section IV, this means that the model determines the evolutionary change between two generations. Figure 1 provides an example of such a causal model, which, if all the causal connections are linear, yields the breeder's equation [see Otsuka (2019*b*) for more details].

In other words, whenever we apply the breeder's equation, we are really assuming that the target population has the causal structure shown in Fig. 1. If this causal assumption is inadequate, the breeder's equation fails to predict evolutionary change correctly. A famous example of this is the evolution of laying date in birds. Laying early in the breeding season typically results in more offspring, and laying date has a heritable basis. Thus the breeder's equation (evolutionary response *R* = heritability *h* ^2^ × selection differential *S*) predicts an evolutionary response towards earlier breeding. In contrast to this prediction, this pattern is often not observed: paradoxically, laying date does not respond to selection (evolutionary response = 0 ≠ heritability × selection differential). It turns out that this is often because the correlation between laying early and reproductive success is actually due to a common third variable, better physical condition due to a favourable environment, which leads to both laying earlier and having more offspring (Price, Kirkpatrick & Arnold, 1988). The observed correlation between laying early and reproductive success is therefore not due to a causal effect of laying date on reproductive success. By including this extra variable and its causal effects, we can re‐parameterise the model and obtain a better estimate for the (lack of) evolutionary response. Hence, to obtain a better prediction and greater insight, a properly defined causal model is in order.

### (2) Expanding the basic causal model for greater insight

**Fig. 2:**
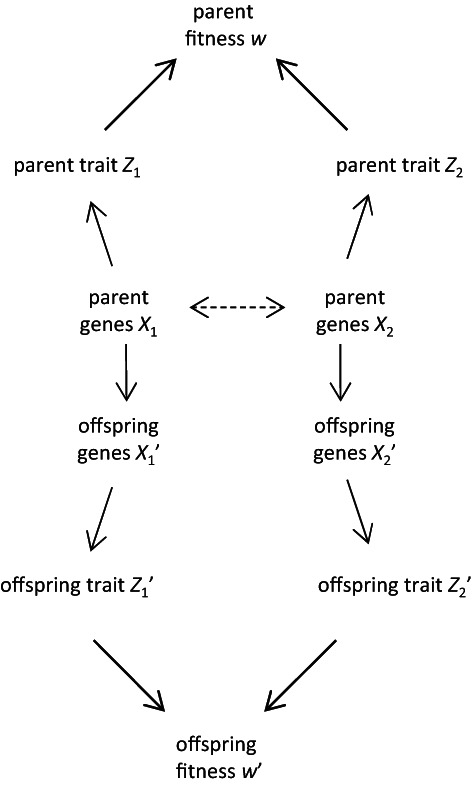
A causal model for evolutionary change involving two traits. Genetic covariance between genes, indicated by a dashed double‐headed arrow, is optional.

Here we argue that adding extra phenotypic variables and causal connections to the basic model will result in a richer view of evolutionary processes and outcomes. We first add one extra trait and turn the above model (Fig. 1 right) into a bivariate selection model (Fig. 2). This could be expanded to even more variables, but for our purposes here two traits are sufficient. The traits of interest are defined by the researcher (decisions on variables and causal connections are up to the person constructing the causal model, depending on the problem at hand). Most often the first trait *Z*_1_ will be a standard phenotypic trait. The second trait *Z*_2_ can be taken to be an additional phenotypic trait of the focal individual (e.g. morphology, behaviour, epigenome; but see Section III.3 for alternative possibilities). Such a causal model captures multivariate selection of the type of Lande & Arnold (1983). Considering multiple traits has two advantages: it allows the user to consider different types of traits at the same time, and it allows for traits to respond to each other. We treat these two aspects in turn below.

## III. TOWARDS A GENERALISED MODEL OF ADAPTIVE EVOLUTION

When thinking about adaptive evolution, we need to focus on two components: what is happening in the parental generation that increases fitness (the adaptation component), and whether this is transferred to the next generation (the evolution component). We treat the transfer component further below (Section III.4). With respect to what happens in the parental generation, countless textbooks, scientific papers and wildlife documentaries have covered the myriad ways in which organisms try to survive and reproduce under the ecological pressures of competition, predation, disease, cold, heat, drought, environmental change and disasters, etc. These can be subdivided according to, for example natural/sexual/social selection, intra‐/interspecific interactions, by trophic level, etc. The classification we follow here is that of Edelaar & Bolnick (2019), since it provides some insights that are used for the generalised model of adaptive evolution that we develop. They argued that adaptation and hence expected fitness is determined by the match between the phenotype and the environment in which this phenotype must operate. In order to improve this match, either the phenotype can be matched to the environment (e.g. *via* growth or phenotypic plasticity), or the environment can be matched to the phenotype (e.g. *via* niche construction or habitat choice): both phenotype and environment can be targets for the organism to achieve greater adaptation [see also Waddington (1957, p. 107), Trappes *et al*. (2022), Child (1997) and Johnson (2007) for a similar classification in biology, economics, and psychology].

This simple observation highlights two important aspects about adaptation (in the sense of increasing expected fitness) which we think are important for a generalised view of adaptive evolution. First, that it is useful to see certain aspects of the environment as extended phenotypes (Dawkins, 1982; Bailey, 2012) when these aspects are changed by the organism and this change potentially affects their fitness (e.g. a bird building a nest). Second, that if what is being optimised by the organism is the phenotype–environment match, then the adaptiveness of a change made to one target (say, the environment) depends on the value of the other target (say, the phenotype), and targets (traits) should therefore respond adaptively to each other (e.g. the size of the nest should match the size of the individual using it). We develop these two points in detail below.

### (1) Extending the phenotype

The term ‘extended phenotype’ was made famous by Dawkins (1982), and has been defined as any change made to the environment (i.e. beyond the integument of the organism; Woods *et al*., 2021) due to the actions of an organism which potentially influences the reproductive success of this organism, intentionally or not. A good example is the nests of birds. These can be highly complex structures which are made by an individual with the aim of increasing its reproductive success (attracting mates, holding and protecting eggs and chicks). Natural selection will therefore favour individuals making the most successful nests, and if nest‐building capacity is heritable, then it will evolve (Perez, Gardner & Medina, 2020). The nest is not part of the organism as we typically recognise it. For example field guides for bird identification do not typically include the nest of every species of bird depicted. On the other hand, nests can be useful for identification and specialised books on birds' nests do exist. Hence, we could see the nest an individual makes as an extended phenotype, and the act of nest building as the development of its extended phenotype [just as Odling‐Smee (2010) called niche construction a developmental process occurring in an ecological context]. The same arguments can be made for other changes made to the environment. Dawkins (1982) focussed on three categories: organisms making physical structures; parasites changing host behaviour; and signallers changing behaviour in recipients. These three categories are all covered by what Edelaar & Bolnick (2019) called ‘adjusting the environment’ (where the environment is abiotic or another organism). However, Dawkins' (1982) categories do not completely encompass the conceptual richness of the term. Edelaar & Bolnick (2019) recognise ‘choosing (selecting) the environment’ (sometimes called ‘organismal selection’; Weiss, 2004) as a second way to alter the environment in order to increase adaptation, and proposed that this process should also be seen as involving changes to the extended phenotype. For example, what if a bird does not build a nest, but chooses a cavity in a tree to lay its eggs. Again, the type of cavity chosen (size of opening, height above the ground, orientation, depth of the cavity, etc.) is due to an action of the organism (its search for and comparison among options and final choice), and likely influences its reproductive success (providing protection against rain, sun, predators, etc.) (Deeming *et al*., 2020). Hence, the chosen cavity could productively be seen as an extended phenotype, just as when the cavity was actually excavated by the bird (e.g. as woodpeckers do). Following that logic, we can then apply these criteria to other aspects of the environment that are chosen by an organism, e.g. a sexual or social partner, an interacting microbe or symbiont (Hurst, 2017), or a type of habitat. As long as these are under the influence of the focal organism and in turn affect its fitness, then those aspects of the environment fulfil the requirements of an extended phenotype and could (if not should) be seen as such. In short, the classification of Edelaar & Bolnick (2019) of the drivers of adaptation into four categories can be used to recognise two groups: when the target for adaptation is the phenotype as we typically understand it then this refers to change in the classical phenotype, and when the target for adaptation is the environment with which this phenotype has to interact, then this refers to change in the extended phenotype, whether this is by adjusting or by choosing the environment. A similar observation has been made by Odling‐Smee *et al*. (2003) when discussing niche construction [see also D'Aguillo, Edwards & Donohue (2019) for germination time in plants as a choice of environment and therefore as an extended phenotype], but here we suggest that the derivation by Edelaar & Bolnick (2019) formally shows that a wider usage of the term extended phenotype is justified and called for.

While we think this wider application of the term ‘extended phenotype’ is a logically consistent conclusion, it does result in some unusual applications of the concept. For example, the environment is typically seen as an external and independent variable to which an organism is exposed, and which it must deal with. This is an important underlying assumption of the field of quantitative genetics, where variation in phenotypes (*V*_P_) is seen as being caused by variation in genotypes (*V*_G_) and variation in developmental environments (*V*_E_), i.e. *V*_P_ = *V*_G_ + *V*_E_ (+ other variance components). This decomposition in causal effects is complicated when the environment in which the phenotype develops is actually (at least in part) under organismal control. This possibility does not invalidate the quantitative genetic approach to understanding phenotypic variation, but it does become important then to identify which aspects of the environment are truly independent external influences, and which aspects of the environment should in fact be seen as extended phenotypes, and therefore could be treated and analysed the same way as classical phenotypes. A good example is the type of habitat an individual is found in. We might find dark grasshoppers occurring on dark substrates and pale individuals on pale substrates, probably because this increases crypsis and therefore decreases predation risk (Edelaar *et al*., 2019). If phenotypic variation in grasshopper colour is analysed, a quantitative genetics approach might infer that grasshopper colour is a phenotypically plastic response to substrate colour (i.e. matching the phenotype to the environment). However, it is also possible that grasshoppers have a fixed colour at birth, but subsequently choose their substrates in order to increase crypsis [i.e. matching the environment to the phenotype – which in fact they do (Edelaar *et al*., 2019; Camacho *et al*., 2020)]. In that case, the substrate on which a grasshopper is found is not an independent external variable, but instead should be approached as an (extended) phenotype of interest, and as the dependent variable of the statistical model. Not taking this alternative into account when using so‐called animal models (Kruuk, 2004; Postma & Charmantier, 2007; Wilson *et al*., 2010) can lead to large differences in value and interpretation of the estimated amount of additive genetic variance and strength of phenotypic plasticity (G. Munar‐Delgado, Y. G. Araya‐Ajoy & P. Edelaar, in preparation; see also Saltz, 2019; Fokkema *et al*., 2021; Gervais *et al*., 2022). Hence, certain aspects of the environment should be seen as organismal traits that are actively changed by organisms because they affect reproductive success, and which therefore might evolve. *A generalised model of adaptive evolution should therefore also apply to the entire set of extended phenotypes*.

### (2) Including that traits can respond to each other

Adaptive evolution *via* natural selection depends on the relationship between traits and fitness. There are many ways to quantify and evaluate this relationship. For example, we could simply estimate the genetic covariance between traits and relative fitness (the ‘secondary theorem of selection’; Robertson, 1968). However, that actually tells us very little about the phenotypic process of selection. For example, a trait could covary genetically with relative fitness yet not causally influence fitness and therefore not be selected at all itself, but just be genetically correlated with a trait that does causally influence fitness. To remedy this and gain more insight, we can analyse the effect of all relevant traits on fitness, and estimate the direct effect of each trait on fitness while statistically taking the correlations between traits into account, *via* multiple regression (Lande, 1979; Lande & Arnold, 1983). The results however are very dependent on which traits have been included, and the interpretation is not intuitive. Consider for example (Arnold, 1983) an extreme situation where variation in morphology (e.g. leg length) only influences performance (e.g. running speed), and that variation in performance in turn influences fitness (e.g. escape from predators). In biological terms, morphology clearly has an effect on fitness, *via* performance. However, a multiple regression model including morphology and performance as explanatory variables for fitness variation would return that there is natural selection only on performance, and not on morphology. This is because the estimated direct selection gradients are measures of selection on traits corrected for all other traits, so on relative morphology (corrected for variation in performance) and on relative performance (corrected for variation in morphology). If morphology only affects fitness *via* its effect on performance, then indeed relative or ‘performance‐corrected’ morphology has no further effect on fitness and is not under direct selection. By contrast, when performance is not included in the model, we find that morphology is in fact under direct natural selection, because the uncorrected morphology indeed does influence fitness. Hence, selection gradient estimates depend on which variables are included in the model, and to what extent these covary. These effects are well known (Kruuk, Merila & Sheldon, 2003; Morrissey, Kruuk & Wilson, 2010), and model formulation and interpretation should be done with care. However, what is less appreciated is that the correlations between variables may not be due to phenomena that are common to the involved traits, like pleiotropy or genetic linkage disequilibrium, or shared developmental environments. Instead, they might arise because of structured, hierarchical effects where one variable affects the other (as in our example where morphology ⇒ performance ⇒ fitness). And since a trait may contribute to fitness *via* multiple pathways, the total causal effect of a trait on fitness may differ from the selection gradient in magnitude or even in sign (Morrissey, 2014), therefore completely changing our interpretation of how selection operates. Such structured, chain‐like relationships, where variables not only directly affect fitness but also have indirect effects *via* other *responsive* variables are likely very common in biological systems.

A specific example and application of this indirect route to fitness variation is found in models of so‐called ‘indirect genetic effects’ (Wolf *et al*., 1998; McGlothlin & Brodie, 2009). These are usually applied to the social interactions between different conspecific individuals. Here the trait values of an actor (e.g. level of aggression) can affect a recipient's fitness in two ways: directly (e.g. killing a recipient's offspring), or indirectly *via* the actor's effect on the recipient's traits (e.g. reducing the recipient's feeding rate). This added indirect route is relevant, because the recipient's trait is now influenced by its own genes and by the genes involved in the trait of the interacting individual (genetic variation in level of aggression), so the occurrence of indirect genetic effects can change the amount of genetic variation in traits and can change the strength and direction of selection. Indirect genetic effects can thereby completely change the evolution of a trait (Wolf *et al*., 1998; McGlothlin & Brodie, 2009). Parental effects models (e.g. Cheverud, 1984; Kirkpatrick & Lande, 1989; Townley & Ezard, 2013) can also be seen to fall under this umbrella of indirect genetic effects: the genotype of parents affects their offspring directly *via* the transmission of their genes to their offspring, but also indirectly when the parental genotype influences aspects of parental behaviour (e.g. food provisioning, defence against predators, construction of nests) which in turn influence offspring phenotypes and fitness. The key element of indirect genetic effects models is that a trait in one individual affects a trait in another individual, i.e. traits are *responsive* to other traits. One could generalise the concept of indirect genetic effects and trait responsiveness to include interactions not only between different conspecific individuals (including relatives) but also between different species, or between nuclear and organellular (e.g. mitochondrial, chloroplast) DNA. Or, as treated above with the performance and morphology example, interactions between traits of the same individual (Morrissey, 2014).

The importance and adaptive value of trait responsiveness can be further understood from the central idea of phenotype–environment match as underlying adaptation. When applying this idea at the trait level, the environment (i.e. the context in which a focal trait has to operate) can be taken to mean different things, including a trait of a conspecific, a trait of a heterospecific, or a trait of the same individual. Fitness increases if the ecological match between two traits is greater. That implies that if an individual makes an adaptive change to one trait, it should be beneficial if that change is tailored to its individual value for the other trait, as this improves the match between the two traits. We therefore would expect that trait responsiveness would evolve to enable such matching. Indeed, all three processes recognised in Edelaar & Bolnick (2019) by which individuals can increase their fitness have such responsive forms, along with non‐responsive forms (see also Trappes *et al*., 2022). Adjusting the phenotype can increase fitness simply because of non‐responsive (canalised) development of the phenotype (e.g. growing larger), which allows the individual to access more resources, escape predators, etc. However, a better adjustment to environmental challenges might be achieved by adaptive phenotypic plasticity, where a single genotype can give rise to different phenotypes depending on environmental or performance cues during development (Snell‐Rood *et al*., 2018; Matthey‐Doret, Draghi & Whitlock, 2020). This is therefore the flexible, environment‐responsive form of adjusting the phenotype. While developmental plasticity was initially viewed as a nuisance to experimentalists (West‐Eberhard, 1989), it is now recognised as a hugely important and frequently adaptive phenomenon with massive demographic, ecological and evolutionary consequences such as enhanced local adaptation, population survival, colonisation of novel habitats, maintenance of genetic variation, etc. (West‐Eberhard, 1989; Miner *et al*., 2005; Chevin, Lande & Mace, 2010; Sultan, 2015; Pfennig, 2021). The responsiveness of traits is therefore of key interest to us.

Similarly, the development of *extended* phenotypes can be responsive, including to phenotypic variation. As an example of choosing the environment, this is now well recognised for habitat choice (Edelaar *et al*., 2008; Akcali & Porter, 2017). Typically habitat choice is thought to be regulated by genetic variation in habitat preferences, e.g. phytophagous insects whose genetically determined taste receptors determine if a host plant will be accepted or not (Jaenike & Holt, 1991). In that case habitat choice is not phenotype responsive but genetically determined, and habitat choice might in fact result in a phenotype–environment mismatch (Patten & Kelly, 2010; Battin, 2004). Compare this to matching habitat choice, which is based on an evaluation of local ecological performance (Ravigné, Olivieri & Dieckmann, 2004; Edelaar *et al*., 2008). Phenotypic variation influences local ecological performance, and hence here habitat choice is phenotype responsive: what is a good matching habitat for one individual (e.g. a dark background for a dark grasshopper) might be a poorly matching habitat for another individual (a dark background for a pale grasshopper; Baños‐Villalba, Quevedo & Edelaar, 2018). *Via* phenotype‐responsive habitat choice (Lowe & Addis, 2019; Camacho *et al*., 2020), individuals will occupy favourable habitats (with similar colours as themselves) and thereby increase their expected reproductive success (*via* greater crypsis). Similar to adaptive plasticity, this phenotype responsiveness may enhance local adaptation, population survival, colonisation of novel habitats, maintenance of genetic variation, etc. (Levins, 1963; Ravigné *et al*., 2004; Edelaar *et al*., 2008; Bolnick & Otto, 2013; Edelaar, Jovani & Gomez‐Mestre, 2017). If mating occurs within habitats, it will also result in assortative mating and (even going beyond the effects of plasticity) reduced gene flow between habitats and locally adapted populations (Maynard Smith, 1966; Diehl & Bush, 1989; Nicolaus & Edelaar, 2018; Edelaar *et al*., 2019). Such phenotype‐responsive and non‐responsive forms are also expected to exist for choosing the environment that involves other aspects, such as a sexual partner: preference might ‘simply’ be driven by genetic variation, or by a more tailored assessment of whether the potential partner provides a good combination with the choosing individual (Mays & Hill, 2004; see also Porter & Akcali, 2018). Overall, trait responsiveness may have highly diverse and potentially large and adaptive consequences, may occur in classical and extended phenotypes, may change the amount of heritable trait variation, and may change the strength and direction of natural selection. This means that focussing on a single trait in isolation is generally insufficient to understand its evolution: just as traits respond to independent, external environmental influences, traits also influence and respond to each other, and this changes their effects on fitness. *A generalised model of adaptive evolution should therefore incorporate that traits can respond to each other*.

### (3) A causal model that includes extended phenotypes and that traits can respond to each other

**Fig. 3:**
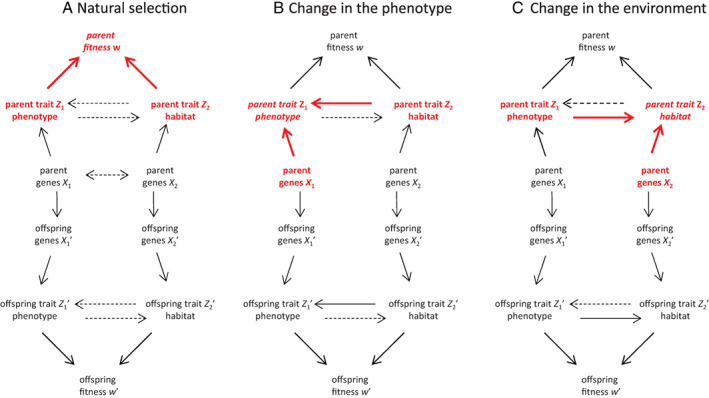
Capturing with a single causal model the different processes that can drive adaptation as identified in Edelaar & Bolnick (2019). Each panel highlights a different aspect of the overall model. The main variables and causal effects involved in each process are indicated in red, and the dependent variable is indicated in italics. Dotted lines are optional. Natural selection (A) is the effect of the phenotype and the environment on fitness [responsiveness of traits and genetic covariance between genes (indicated by a double‐headed arrow) are optional]. Change in the phenotype (B) involves the development of the organism's phenotype due to its genotype, and any additional responsive change due to environmental inputs (i.e. phenotypic plasticity; likely additional, organism‐independent environmental inputs are not depicted). Symmetrically, change in the environment (C) (choice and adjustment of the environment, *cf*. Edelaar & Bolnick, 2019) involves the development of the organism's environment due to its genotype, and any additional responsive change due to phenotypic inputs (i.e. a form of plasticity; likely additional, organism‐independent environmental inputs are not depicted).

We incorporate these two considerations in our next version of the causal model (Fig. 3). First, we incorporate that traits can respond to each other. This involves including an arrow from one phenotype variable to another, but what this actually represents biologically depends on what these variables are. If a regular phenotypic trait responds to another regular phenotypic trait, then the causal model captures the situation of causally covarying traits, as treated by Morrissey (2014). The second trait *Z*_2_ can also be taken as a trait of another conspecific individual, which then produces a model of indirect genetic effects (e.g. Araya‐Ajoy *et al*., 2020). Or, as per our second consideration of incorporating the entire set of extended phenotypes, it can be taken to be any other (extended phenotypic) aspect of the environment, as introduced above. If we take as the second trait an aspect of the environment under control of the organism, then the bivariate model can represent the simultaneous evolution of the classical phenotype and the extended phenotype of an organism. With this interpretation, each of the adaptive processes distinguished in Edelaar & Bolnick (2019) can be represented by corresponding causal arrows (Fig. 3).

The causal model of Fig. 3 captures that adaptation is the phenotype–environment fit, and that greater adaptation might occur if either of these two components responds to the other.

### (4) Inheritance can be non‐genetic too, and this should be expected to occur

We now discuss the component of transfer between generations. As mentioned before, there are several distinct processes by which individuals can change their phenotype or extended phenotype. If these processes are performed adaptively, individuals can expect higher reproductive success and leave more offspring. However, these offspring are often very different from the adult parental phenotypes, generally much smaller and less developed (e.g. a plant seed, a fly larva, a human baby). Fortunately, these offspring have a firm basis with which to achieve successful reproduction themselves. First, they receive copies of parental genes. Since their parents managed to reproduce successfully, it is likely that these genes also help offspring to develop, respond and function in a manner that provides a good phenotype–environment match. For this reason the genome is sometimes seen as a way to transfer information across generations about how to deal with the environment (e.g. Frank, 2009; Griffiths, 2017). This genetic way of influencing organismal performance across generations applies to all three individual‐level processes (Edelaar & Bolnick, 2019) to increase fitness (e.g. a bird receives genes for developing wings and for knowing where and how to build a nest). Because some genetic variants are better than others at achieving a phenotype–environment match they leave more offspring, i.e. there is natural selection on the genetic variants involved in the processes providing this match. Since the genetic variants are heritable, these beneficial genetic variants will increase in the next generation, and this is the standard way in which natural selection drives adaptive genetic evolution.

However, for an offspring to function well, there is no requirement that adaptive parental traits are only transmitted genetically. For example, if an offspring can somehow copy (learn) an adaptive parental behaviour (e.g. where and how to build a nest), then this will also increase the offspring's expected fitness. Hence, receiving (inheriting) beneficial parental traits non‐genetically is a second way by which offspring are being prepared or preparing themselves to deal with the ecological challenges they face.

Such non‐genetic inheritance is probably more common than typically assumed, and has now been found to occur *via* a diversity of mechanisms (Danchin *et al*., 2011, 2019*b*; Rechavi, Minevich & Hobert, 2011; Bonduriansky, 2012; Dias & Ressler, 2014; Bošković & Rando, 2018; Bonduriansky & Day, 2018; Adrian‐Kalchhauser *et al*., 2020; Edelaar *et al*., 2021; Anastasiadi *et al*., 2021). These include the transmission *via* gametes of molecular epigenetic marks on DNA, small RNAs, cytoplasmic and structural properties of cells, hormones, and nutrients. Transmission *via* other parent–offspring interactions (e.g. parental care or simply proximity) of nutrients, hormones, antibodies, behaviours, symbionts, and microbiomes is also possible. The concept of non‐genetic inheritance can even be applied to the transmission of extended phenotypes (the ‘envirotype’; Mameli, 2004) *via* so‐called environmental (ecological) inheritance [see Pontarotti (2020) for a critical review]. For example, offspring may inherit the physical structures (nests, burrows etc.) that their parents built or owned, and the same can be said of food stores, territories, or simply habitats and geographic locations, since offspring are often born where their parents resided. Hence, non‐genetic inheritance is really a collection of very different types of inheritance [molecular epigenetic inheritance, molecular and cellular structural inheritance, behavioural inheritance (learning), ecological inheritance] which share the feature that the relevant variation that is inherited is not genomic DNA sequence variation (genetic inheritance). Some of these types have been long known (e.g. learning and copying, as in the field of cultural evolution), but others are more recent (small RNAs) and yet others might be discovered in the future. The overall effect of non‐genetic inheritance is that it can cause resemblance between parents and offspring, and can do so for a large set of phenotypic and extended phenotypic traits.

Hence, this alternative cause to parent–offspring resemblance may allow offspring to achieve a similar phenotype–environment match as their parents, without having to start from scratch using only their genotype. Natural selection will therefore favour the evolution of the mechanisms involved in non‐genetic inheritance, if they increase fitness (e.g. Rivoire & Leibler, 2014; English *et al*., 2015). This evolution likely involves the genes that are involved in non‐genetic inheritance. For example, if it is advantageous for offspring to copy parental behaviours, then genetic variants which do this more or better will increase in the population, resulting in greater non‐genetic inheritance of behaviour.

This non‐genetic transmission of phenotypic and extended phenotypic traits across generations can change evolutionary dynamics (Danchin *et al*., 2011, 2019*b*; Day & Bonduriansky, 2011; Townley & Ezard, 2013; Mesoudi *et al*., 2013; Bošković & Rando, 2018; Bonduriansky & Day, 2018; Adrian‐Kalchhauser *et al*., 2020; Anastasiadi *et al*., 2021). First, it will change the selective pressures to which organisms are exposed: depending on variation in their phenotype (e.g. learnt feeding behaviours) or extended phenotype (e.g. nests or food reserves produced by ancestors), a certain external environmental pressure (e.g. a cold spell) will have different effects on the reproductive success of individuals and their traits (e.g. body size, or ability to generate heat). Second, since natural selection is only a consequence of how organisms perform ecologically, natural selection will also occur for non‐genetic trait variants and change their frequency in the parental generation (El Mouden *et al*., 2014). Then, to the extent that non‐genetic traits are transmitted between generations, natural selection will cause a change in the frequency of this non‐genetic trait in the next generation too. In other words, since natural selection is blind to the causes of trait variation, the trait will evolve whether inheritance is genetic or non‐genetic: adaptive evolution *via* natural selection is also blind to the causes of inheritance (Mameli, 2004). We later discuss whether such a between‐generation change in the non‐genetically heritable traits of a population should be seen as evolution [since for some people evolution is exclusively defined by genetic change, but we already note here that many quantitative genetic models and equations (e.g. the breeder's equation, Fisher's theorem, the Price equation) actually do not make (or need to make) that restriction] or perhaps as non‐genetic evolution [good examples of this are cultural evolution (El Mouden *et al*., 2014; Creanza, Kolodny & Feldman, 2017; Whiten, 2019) and changes in epigenetic molecular states (Cortijo *et al*., 2014; Gahlaut *et al*., 2020)]. But for now, for the reasons mentioned above, we postulate that *a generalised model of adaptive evolution should incorporate that traits can be inherited genetically as well as non‐genetically*.

**Fig. 4:**
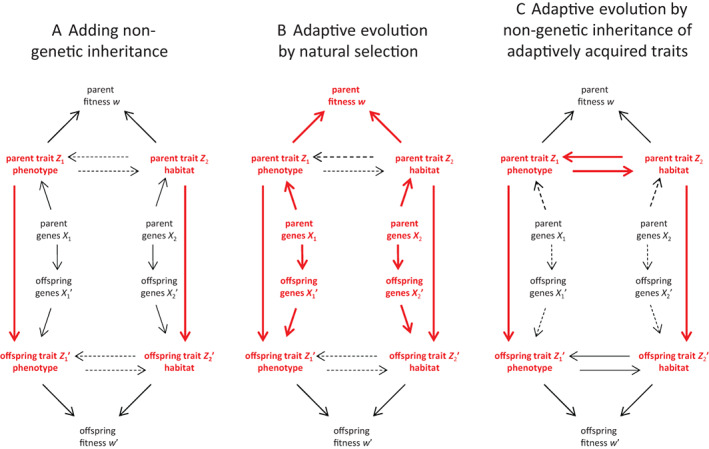
Adding non‐genetic inheritance to the causal model of Fig. 3 to make it even more general (A). This addition identifies two very distinct forms of adaptive evolution (compare B with C). First, adaptive evolution can be driven by natural selection (B), when traits (classical or extended phenotypes) increase fitness and are transmitted to offspring. Importantly, whether this transmission is achieved genetically or non‐genetically is inconsequential for its occurrence and operation, since fitness depends on phenotypes (and not genotypes). Second (C), adaptive evolution can be driven by the non‐genetic transmission to offspring of parental responses (acquired traits, e.g. parents move into a different habitat and in response acquire a new parental behaviour, which is subsequently copied by offspring), assuming this parental response itself is adaptive. For this, transmission needs to be non‐genetic because changes in phenotypes are not registered in the genotype (as far as we know). Red variables and arrows highlight the key elements of each panel. Dotted arrows indicate causal effects that are optional.

By not going into mechanistic detail (which is done to some extent for the genetic inheritance pathway), this inclusion is simply done by drawing a causal arrow between the parental and offspring trait (Fig. 4A): somehow offspring resemble parents due to inputs that are independent of the genotype they receive from their parents (e.g. copying in the case of a behavioural phenotype, or imprinting in the case of the habitat). [Some non‐genetic epigenetic molecular marks are non‐responsive and long‐lived, so basically operate as genetic alleles in terms of causal effects (Cortijo *et al*., 2014; Banta & Richards, 2018; Sentis *et al*., 2018; Thomson *et al*., 2018; Gahlaut *et al*., 2020). For simplicity these are included here in the genetic inheritance pathway.]

### (5) The consequences of non‐genetic inheritance

Adding a route of non‐genetic inheritance to the causal model of Fig. 3 (as done in Fig. 4A) has two important consequences. First, it generalises that natural selection can drive adaptive evolution independent of the exact details of inheritance, whether genetic or non‐genetic (highlighted in Fig. 4B), because natural selection operates *via* phenotypes (not genotypes), and is therefore blind to the causes, including transmission, of phenotypic variation. Hence, natural selection can drive the evolution of a trait that is fully non‐genetically inherited, for example a learned behaviour or an imprinted habitat, when this trait is (directly or indirectly) related to fitness.

Second, as we will argue below, non‐genetic inheritance opens up the possibility that traits that respond adaptively to other variables in the parental generation (i.e. are adaptively acquired) can be transmitted to the next generation (highlighted in Fig. 4C). Since the causal pathway does not involve parental fitness (as it does in Fig. 4B), this is a distinct, second type of adaptive evolution (i.e. increasing the mean fitness across generations). In other words, it is a driver of adaptive evolution that is independent of current natural selection (fitness variation among parents is not the driver of the process).

## IV. EVALUATING OUR GENERALISED MODEL IN THE CONTEXT OF THE PRICE EQUATION

Above we have shown ways to model various evolutionary mechanisms in terms of bivariate causal models. The next task is to identify whether and how these causal pathways affect evolutionary change. Evolution is a heritable change in a phenotypic or genotypic distribution, and we focus here on the change of its mean. This change in the trait mean is often usefully described by the Price equation (Price, 1970, 1972; Frank, 2012*a*):

(1) $$\Delta \bar{Z_{i}}=\;\mathrm{Cov}\left(w,{Z_{i}}^{\prime }\right)\;+\;E\left({Z_{i}}^{\prime }-Z_{i}\right)$$

where the left‐hand side $\Delta \bar{Z_{i}}$ denotes the change of the mean of trait *Z*_*i*_, with *i* = {1, 2} in our model, between two time steps: in our model the parental and offspring (indicated by a prime symbol) generation. The variable *Z*_*i*_ *’* on the right‐hand side is the average offspring trait value of parent *i*, and *w* is the relative fitness of the parent, i.e. the offspring count divided by the mean offspring count of the population. The equation divides the evolutionary change into two components. The first term is the covariance term *Cov*(*w*, *Z*_*i*_ *'*) that measures the statistical association between parents' relative fitness *w* and the average phenotypic value of their offspring. The second term, *E*(*Z*_*i*_ *' – Z*_*i*_), is the expected mean extent to which the offspring's average phenotype differs from their parent's phenotype (so the difference is first calculated for each parent, and then averaged over all parents).

The two terms in the Price equation, the covariance and expectation, are often interpreted to represent natural selection and transmission bias, respectively. This interpretation, however, does not always hold, for in some cases the covariance term confounds selection and other factors [for example, stochastic variation (as in genetic drift) also causes covariance (Rice, 2004; Okasha & Otsuka, 2020)]. For this reason, evolutionary factors (including most notably natural selection) should be defined in terms of causal pathways, not just by their statistical outcomes: for instance, natural selection *for* a given phenotype or genotype should be understood as their causal effect on fitness (Sober, 1984). This has some important consequences. First, natural selection may affect both the covariance and expectation terms of the Price equation. Second, and more importantly for the following discussion, adaptive change, which is defined as an increase in the population mean fitness over generations, is conceptually distinguished from natural selection. Natural selection, i.e. a phenotypic or genetic contribution to fitness, is certainly one way to raise the population mean fitness (Fisher, 1930). But that is not the only way, as we will discuss below.

To evaluate evolutionary factors quantitatively, then, one needs to bridge between the Price equation and the above causal models. This bridge is provided by Wright's method of path analysis (Wright, 1934). Below we analyse how our causal assumptions affect the covariance and expectation terms of the Price equation and give rise to two different evolutionary processes.

### (1) Effects in the covariance term

#### (a) Trait responsiveness

First, the responsiveness of a trait can change the covariance term *Cov*(*w*, *Z*_*i*_ *'*). According to Wright's method of path analysis (Wright, 1934), the covariance of two variables is determined by causal connections between them. Specifically, any causal paths between two variables that do not contain colliding arrows (such that → *X* ←) contribute to their covariance, and thus are called *active*. Hence by identifying such active paths between parental fitness *w* and offspring phenotype *Z*_1_ *'* (or alternatively *Z*_2_ *'*, depending on which trait is our focal trait), we can analyse the ways in which the causal mechanisms introduced above affect the covariance part of the Price equation. The path of standard genetic evolution by natural selection on a trait (Fig. 3A) (e.g. *w* ← *Z*_1_ ← *X*_1_ → *X*_1_ *’* → *Z*_1_ *’*) is just one among many ways to yield adaptive evolution. For example, if a phenotype responds to heritable habitat variation that affects fitness (as in Fig. 3B), this introduces an extra active pathway (e.g. *w* ← *Z*_2_ ← *X*_2_ → *X*_2_ *'* → *Z*_2_ *'* → *Z*_1_ *'*) and additional variance through which the focal phenotype is affected by natural selection. In such cases evolutionary change is determined not just by the additive genetic variance for trait 1 but also by the heritable variance in trait 2, so the total amount of additive heritable variance is larger. This could result in greater evolutionary change. However, the consequences for the covariance term *Cov*(*w*, *Z*_*i*_ *’*) are more complex: depending on the sign and size of the coefficients of all causal paths, it may increase or decrease. In addition, responsiveness of traits also can have the result that the focal trait is not the only trait that evolves. For example, if a phenotypic trait affects an aspect of the environment as an extended phenotype (as in Fig. 3C), this creates an active path between the parental fitness and offspring habitat (*w* ← *Z*_1_ ← *X*_1_ → *X*_1_ *'* → *Z*_1_ *'* → *Z*_2_ *'*), which means that the habitat itself may coevolve and respond indirectly to natural selection on the phenotype (in addition to any direct effect of *trait Z*_2_ *habitat* on fitness). In summary, the responsiveness of traits can severely alter the evolutionary consequences of variation in fitness [similar to models of indirect genetic effects (Wolf *et al*., 1998; McGlothlin & Brodie, 2009)]. Ignoring this responsiveness thus may result in a biased view, which is why we included it in our model.

#### (b) Non‐genetic inheritance

Second, non‐genetic inheritance creates additional active pathways (e.g. *w* ← *Z*_1_ → *Z*_1_ *'*) through which organisms can respond to selection (Fig. 4). One salient feature of this type of trait change is that its magnitude is determined by not just genetic but also environmental variance. This is because the strength of an active pathway (i.e. its contribution to the covariance) is determined by, along with path coefficients, the variance of its source. While genes serve as the only source in conventional genetic inheritance, the source of the pathway mediated by non‐genetic inheritance is parental phenotype. This means that all components of phenotypic variance, either genetic or environmental, contribute to change. Hence with non‐genetic inheritance, adaptive phenotypic evolution may occur without any change in the underlying genetic distribution, or even in the absence of genetic variation (as mentioned in Section III.5). This is because fitness variation is caused by phenotypes first (as also shown by *w* ← *Z*_*i*_), and only indirectly, and not necessarily so, by underlying genetic variation (as shown by *w* ← *Z*_*i*_ ← *X*_*i*_). Depending on the strengths of the coefficients and the variance of the variables involved in all relevant pathways, the covariance term (*w*, *Z*_*i*_ *’*) may increase or decrease when non‐genetic inheritance is added. Hence, non‐genetic inheritance of traits can alter the evolutionary consequences of variation in fitness. Ignoring non‐genetic inheritance thus may result in a biased view, which is why we included it in our model.

### (2) Effects in the expectation term: non‐genetic inheritance enables the transmission of adaptive responses as a second driver of adaptive evolution

The added mechanisms of our causal model do not only affect the covariance term of the Price equation. Even more importantly, trait responsiveness and non‐genetic inheritance combine also to affect the expectation term E(*Z*_*i*_ *' – Z*_*i*_). This is because the parental phenotype *Z* may directly affect the offspring phenotype *Z'*, and by doing so introduce a so‐called ‘transmission bias' at the phenotypic level (not a bias *in* transmission, but a bias *caused by* transmission) (El Mouden *et al*., 2014; Otsuka, 2015). For example, let *Z*_2_ be a binary environmental variable which indicates the presence of a new nutritious prey type (*Z*_2_ = 1 for present; *Z*_2_ = 0 for absent). When the prey type first appears in the population, some individuals learn by themselves to use this prey type (*Z*_1_ = 1). If every individual learns to feed on this prey type by itself with probability *b*, then the average phenotype in any generation will be *b* (=*b × N/N*), and *E*(*Z*_1_ *'* − *Z*_1_) = *E*(*b – b*) = 0. In other words, there is no net change in phenotype across generations due to learning (only in the first generation, when new prey first appeared), and only a proportion *b* of all individuals feeds on this advantageous prey type (of course this proportion might increase over generations due to natural selection, see Fig. 4B). However, if offspring also may learn to feed on this prey from their parents, there is a non‐genetic inheritance *Z*_1_ → *Z*_1_ *'*. Assume that learning from parents happens with probability *m*. Then the probability of eating the new prey in offspring is the sum of them learning it from parents that previously learned by themselves (*m* × *b*), and learning by themselves in case they did not learn it from their parents already (*b* × (1 – *m* × *b*)), so *b + b*(*m – m* × *b*). This will bias upwards the frequency of type *Z*_1_ = 1 individuals in the offspring generation because of the added effect of parent‐to‐offspring transmission (i.e. transmission bias): *E*(*Z*_1_ *'* − *Z*_1_) = *b + b*(*m* − *m* × *b*) − *b* > 0, i.e. the average phenotype now *does* change across generations. Moreover, with non‐genetic inheritance, the learned feeding behaviour of parents, grandparents, great‐grandparents, etc. can now all be cumulatively passed down to the next generation(s), and feeding on the new prey may even become a fixed trait in the population because of non‐genetic inheritance (compared to a proportion *b* without non‐genetic inheritance). Thus the combination of trait responsiveness (here: learning to feed on a prey type available in the environment) and non‐genetic inheritance allows by itself for trait change across generations, without currently acting natural selection as part of the driving mechanism. If the responsiveness of traits increases expected fitness in parents (i.e. it is adaptive), then non‐genetic inheritance provides organisms with a means to pass on this adaptation to their offspring and thereby also increase their offspring's expected fitness.

## V. DISCUSSION

If our causal modelling approach is to be heuristically useful, it should shed some light on the three examples introduced in Section I.1 of biological phenomena which appear not to fit comfortably into mainstream evolutionary theory. We therefore now reconsider these three examples as support for our approach, as examples of how to apply the causal model to specific situations, and as support for the added insight this could provide.

### (1) Using our causal modelling approach to understand certain biological phenomena in the context of evolutionary theory

#### (a) How do the latest data on molecular epigenetics fit into mainstream evolutionary theory?

As mentioned in Section I.1.*a*, a number of molecular epigenetic changes are now known to be transmitted from parent to offspring generations, and these can be involved in adaptive phenotypic plasticity. This phenomenon is captured by Fig. 4C, if we take the molecular state(s) of interest as one trait, and the environment causing changes in these as the other trait. The horizontal arrows indicate the responsiveness of a trait to another trait, here the molecular state to some environmental condition. This is essentially phenotypic plasticity, and these molecular marks play an important and well‐known role in within‐generation development (Danchin *et al*., 2011, 2019*b*; Bonduriansky & Day, 2018; Adrian‐Kalchhauser *et al*., 2020). Subsequent parent–offspring transmission is indicated by the vertical arrow capturing non‐genetic inheritance. The combination of these two steps allows for the between‐generation transmission of parental responses we discussed above, something that is thought not to be possible with genetic transmission. Our model then shows that the transmission of parental responses is dependent on the existence of non‐genetic inheritance. Note however that this does not imply that non‐genetic inheritance always involves the transmission of adaptively acquired traits. First, the acquired traits may not be adaptive (this can be seen in the causal graph, since the relevant paths do not include fitness). Many developmental problems or other maladaptive traits (e.g. parasites) can also be acquired and transmitted non‐genetically (Wang *et al*., 2017; Bonduriansky & Day, 2018). In that sense evolution *via* parental responses is also different from evolution *via* natural selection, which simplistically speaking (keeping everything else constant) always results in adaptation. (See Section V.3. for more on whether acquired traits are expected to be adaptive). Second, some non‐genetically inherited traits are (virtually) not responsive, as in Fig. 4B. For example, there are epigenetic molecular marks with transition rates which are almost equally low as genetic mutation, and these basically have dynamics that are hard to distinguish from genetic variation (Cortijo *et al*., 2014; Banta & Richards, 2018; Sentis *et al*., 2018; Thomson *et al*., 2018; Gahlaut *et al*., 2020). For these to evolve adaptively, the pathway involving natural selection (Fig. 4B) is the relevant one. In conclusion, non‐responsive epigenetic marks can be nicely addressed with mainstream gene‐centred evolutionary models, but to understand the dynamics of responsive epigenetic marks we can benefit from using generalised models such as the one we present herein.

#### (b) Is niche construction an evolutionary process?

It has been stated that ‘Niche construction should be regarded, after natural selection, as a second major participant in evolution’ (Odling‐Smee *et al*., 2003, p. 2). Others have responded that ‘Niche construction, like all environmental change, can cause evolutionary processes to occur, but this does not make it an evolutionary process itself. Of the different evolutionary processes (e.g. natural selection, genetic drift, mutation, and migration) only natural selection can explain adaptation’ (Scott‐Phillips *et al*., 2014, pp. 1234–1235). So, is niche construction an evolutionary process resulting in adaptation or not? We think our causal model can help to clarify this debate. We can take niche construction as a change in one of the traits in our model where this trait is some aspect of the environment (so *Z*_2_ and *Z*_2_ *'*), like the size of some physical construction (e.g. a termite mound or a bird's nest). We see that this trait potentially receives several inputs (Fig. 4): genetic input, input from another (phenotypic) trait, and non‐genetic input from parents.

Let us first focus on the genetic input. If this is the only input received, then the environmental aspect of interest really behaves as any other phenotypic trait (niche construction is comparable to development), and may also evolve *via* natural selection (Fig. 3A). It can affect fitness and affect other traits (the outgoing arrows in e.g. Fig. 3B), but that is a common property for any organismal trait. Hence, here niche construction is a cause of evolution because it changes the evolutionary outcome. But this is not a special property, it has this in common with any environmental change, for example drought causes the evolution of bill size in Darwin's finches (Grant & Grant, 2002). With only genetic input, the effect of niche construction on evolution operates *via* natural selection. Therefore, in our view in that case it is not an evolutionary process, because it does not have an *independent* effect on the evolutionary outcome – it just changes the outcome of other evolutionary processes. This type of genetically determined niche construction is the one treated by Dawkins (1982) with his extended phenotype concept.

Let us now focus on the input from another trait, e.g. body size (as in Fig. 3C). This effect is in causal terms identical to phenotypic plasticity, except that the affected trait is not a classical phenotypic trait but an extended phenotype, i.e. some aspect of the environment. For lack of a better term, we have tentatively called this ‘environmental plasticity’ when there is differential development of an organism's environment in response to phenotypic variation (i.e. the logical reverse of phenotypic plasticity; G. Munar‐Delgado, Y.G. Araya‐Ajoy & P. Edelaar, in preparation). This changed environment could again affect the development of other traits and fitness, which again change selection pressures and possible constraints, but again it just changes the outcome of other evolutionary processes (just as phenotypic plasticity is well known to do; West‐Eberhard, 1989; Miner *et al*., 2005; Chevin *et al*., 2010; Sultan, 2015; Pfennig, 2021). So also in this case niche construction is a cause of evolution but we do not see niche construction as an evolutionary process because it has no independent effect on trait change. Note however that the responsiveness of extended phenotypes has been greatly neglected [but see the example on habitat choice in Section V.1.*c*, and Woods *et al*. (2021) for some examples], yet the impact on evolutionary outcomes might be as consequential as for regular phenotypic plasticity. This is therefore a rich area for further theoretical and empirical development.

Finally, let us look at the parental input (Fig. 4A), i.e. the arrow from the parent to the offspring reflecting non‐genetic inheritance. This is where things get even more interesting. The parental input means that the environmental trait is directly transmitted to the offspring generation, also known as environmental (or ecological) inheritance (Mameli, 2004). So a termite queen might inherit the mound of her mother, or a bird might inherit the nest or territory of its parents. As we saw earlier, this means that this aspect of the environment can evolve in two ways. First, *via* natural selection (Fig. 4B). If the properties of a bird's nest affect fitness (and nests hold eggs and chicks, so this is quite likely), then nests providing greater fitness are more likely to be transmitted to offspring, resulting in the adaptive evolution of bird nests (Perez *et al*., 2020). Nonetheless, also here niche construction (nest building) is not an evolutionary process – it is still natural selection that drives adaptation. Second, non‐genetic inheritance allows for the inheritance of parentally acquired traits (Fig. 4C). For example, if nests are adjusted in response to some additional variable (a phenotypic trait like body size or an environmental trait like cold, captured by a horizontal arrow), and this change in the parental generation is subsequently transmitted to the offspring generation, then nests have evolved. Here the driving force is really the response of the individual to other variables, i.e. responsive or plastic niche construction, and it has an independent effect on the composition of a heritable trait of the population, so we can see the developmental step as an evolutionary process. If this response is adaptive (larger nests for larger individuals, warmer nests for lower temperatures; Deeming *et al*., 2012), then this evolutionary change is also adaptive.

In conclusion, an analysis with the help of our causal model shows that the answer to the question ‘is niche construction an evolutionary process?’ does not have a singular ‘yes’ or ‘no’ answer. When natural selection is the focal evolutionary process, then a developmental process like niche construction ‘only’ provides the trait variation, and does not constitute an independent evolutionary process. However, niche construction (and other developmental processes) is an evolutionary process when it is responsive and the relevant evolving environmental trait is non‐genetically inherited, because then niche construction has evolutionary effects (i.e. causes a heritable change in the trait distribution) independent of any current natural selection or other evolutionary processes (mutation, migration) also occurring. One could therefore say that opponents in this debate were all correct in their statements, but only partially, and the reality is slightly more complex: niche construction both is and is not an evolutionary process – it depends on the context. And in our view, it is an evolutionary process specifically and only for the constructed trait (not for any other trait of the organism), but it can be a cause for the evolution of other traits. This complexity should not be ignored, and our causal modelling approach allows people to address this complexity.

#### (c) Is local adaptation via habitat choice also adaptive evolution?

We briefly repeat the problem. On the one hand, individual habitat choice can lead to local adaptation of populations in heterogeneous environments, the same outcome as for divergent natural selection. On the other hand, remarks such as ‘Of the different evolutionary processes (e.g., natural selection, genetic drift, mutation, and migration) only natural selection can explain adaptation’ (Scott‐Phillips *et al*., 2014, p. 1234) are very common in the literature. The conundrum is therefore that individuals may move to places where they perform better and can thereby form locally adapted populations, but this may happen even in the absence of any differential mortality and reproduction between different phenotypes, i.e. in the absence of current natural selection. One solution to this conundrum is that the cited remark receives a qualifier about timescales: at a different scale of explanation the reason for adaptation will always be natural selection, since it is the first evolutionary process of adaptation that arose when life first originated and ultimately this explains the evolution of adaptive dispersal [e.g. Rausher (1984); see Section V.6 for more on this point]. But that does not solve how habitat choice creates adaptive population divergence in the absence of currently acting natural selection. We think another solution lies in considering what it actually is that evolves.

As outlined in Edelaar & Bolnick (2019), adaptive evolution is not the evolution of the phenotype, but the evolution of an improved phenotype–environment match, since that match influences fitness. This is also reflected in Kawecki & Ebert (2004), who discuss how local adaptation should be defined and measured, and their approach basically consists of addressing local adaptation from the point of view of the individual (‘is its fitness higher locally than elsewhere?’) and from the point of view of the location (‘are local individuals fitter than non‐local individuals?’). This necessity to look at both aspects stems from the fact that adaptation is a greater phenotype–environment match, so both components need to be included in our evolutionary evaluations. Typically the focus is on the phenotype component, but for habitat choice it is on the environment component.

In some cases we can treat the environment as homogeneous and not amenable to change by the organism, and in those cases the only possible improvement in match is by change in the phenotype, e.g. when peppered moths (*Biston betularia*) evolved a darker colour to increase crypsis on polluted tree trunks (Cook, 2003). This ‘phenotypes evolve’ is perhaps the mainstream view. However, in other cases the environment can be chosen or adjusted, and it is perfectly possible to improve the match by changing the environment while keeping the phenotype constant, e.g. when grasshoppers of different colours choose to perch on matching backgrounds to increase crypsis (Edelaar *et al*., 2019; Camacho *et al*., 2020). Niche construction is an example of this change to the environment, and so is habitat choice. The analysis of the evolutionary relevance of habitat choice is therefore similar to the analysis of niche construction above.

Just as for any focal trait in our causal model, the chosen habitat has three inputs (Fig. 4A). First, if habitat choice is due to some genetic input only, the classical model of evolution by natural selection can be used (Fig. 3A). However, the trait that is evolving in our causal model is not the genetic basis to habitat choice, it is actually the chosen habitat – *that* is the relevant trait that influences fitness, and that is the one that is exposed to and responds to natural selection, just as when natural selection acts on peppered moth colour. The evolution of habitat‐preference genes is only an indirect consequence of natural selection on habitat use, because genes happen to cause this variation in habitat use (Jaenike & Holt, 1991). Viewing aspects of the environment that are influenced by individuals as traits that can evolve fits with other approaches. Dawkins (1982) discussed the evolution of extended phenotypes that can evolve due to genetic changes, although he did not extend it to choice and adjustment of the environment. Comparative studies may focus on analysing the evolution of traits like host plant use or nest location, typically assuming these are genetically determined aspects of the environment.

Second, habitat use may also be due to non‐genetic inheritance (Fig. 4A). One mechanism for this is imprinting by young individuals on the habitat used by their parents (Davis & Stamps, 2004; Akcali & Porter, 2017), potentially even without any genetic input. The habitat used may then still evolve *via* natural selection, if habitat affects fitness (Fig. 4B). Other mechanisms of non‐genetic inheritance may also be considered. For example, the breeding location of offspring is often similar to that of parents, e.g. due to limitations in mobility, or because of other reasons resulting in natal philopatry. Hence, location is quite heritable (*cf*. nationality in humans). Treating location as an organismal trait (even though its ‘control’ over it is not obvious at first) is in line with comparative biogeographic and phylogeographic studies that investigate the evolution of distribution ranges (as an emergent higher‐level property of individual locations).

Finally, habitat use may respond to other traits of the organism (Fig. 4C), for example its phenotype. Adaptive, phenotype‐dependent habitat choice is known as matching habitat choice (Ravigné *et al*., 2004; Edelaar *et al*., 2008), where a habitat is chosen by an individual because it increases the (perceived) phenotype–environment match; grasshoppers choosing colour‐matching surfaces is a good example (Camacho *et al*., 2020). This is similar to phenotypic plasticity but then acts in the reverse direction. It is this responsive quality that sets matching habitat choice apart from habitat choice due to preference alleles or imprinting (Akcali & Porter, 2017), for two reasons. First, the adaptive element is intrinsically part of the mechanism: a habitat is chosen because it is perceived as increasing phenotype–environment match. This is not true for habitat choice due to preference alleles or imprinting: it is quite possible that more matching habitats are available given an individual's phenotypic characteristics, but this is just not considered by these mechanisms. Second, the chosen habitat is not heritable just because of the response (the red arrow *Z*_1_ → *Z*_2_, highlighted in Fig. 4C), whereas it is heritable for habitat choice due to preference alleles (the arrow *X*_2_ → Z_2_) and imprinting (the arrow *Z*_2_ → *Z*_2_ *'*). This means that matching habitat choice only has evolutionary consequences if there is also non‐genetic inheritance (the same as for phenotypic plasticity). Since many organisms show some degree of philopatry at the spatial scale of habitat choice, this may not be uncommon (i.e. non‐genetic heritability is larger than zero). Hence, matching habitat choice is another example of how parental responses (acquired traits) might be transmitted to future generations if it combines with non‐genetic inheritance, but an example where it is the chosen habitat that is the trait that evolves. In conclusion, local adaptation due to habitat choice is also adaptive evolution, but it is evolution of the chosen habitat.

#### (d) What general conclusions can we draw from these three examples?

First, even though the three examples from the introduction seemed to be about very different biological topics and about different ways to achieve higher fitness (in fact they represent one of each of the three individual‐level processes increasing fitness according to the classification in Edelaar & Bolnick, 2019), it turns out that they can all be meaningfully investigated with the same causal model. This supports that the causal modelling approach, and the specific model we propose, can be used generally to achieve a better understanding of evolutionary change, when the focus is on the processes and mechanisms that drive evolution. Second, in each of the three examples, the responsiveness of traits in combination with non‐genetic inheritance enables a different, second kind of adaptive evolution. This supports that this distinct kind of adaptive evolution (which does not involve current natural selection to achieve it) may be found in a wide range of traits and biological systems (e.g. microbiomes), if we care to see it. Third, it suggests that recognising this second route to adaptive evolution helps make theoretical sense of phenomena and observations that otherwise are hard to fit into standard evolutionary theory.

### (2) A pluralistic view on adaptive evolution

Our model shows that a broader view of adaptation and evolution may help to explain some puzzling phenomena. Textbooks usually define evolution as change in allele and/or genotype frequencies across generations, and mention natural selection as the only driver of adaptation. However, our model shows that, from a theoretical point of view, both evolution and adaptation can be achieved through different pathways and drivers. This requires some revision of the traditional definitions of evolution and adaptation. We are aware that, sometimes, discussions about definitions can be unproductive (and for that reason we complemented our view with a causal model). At the same time, great advances in science have also consisted in the formulation of a new meaning of some basic concept (such as time or space in physics). Some people may argue that evolution is ‘by definition’ genetic (Day & Bonduriansky, 2011; Futuyma, 2017), but many basic evolutionary equations (e.g. the breeder's equation, Fisher's theorem, the Price equation, other quantitative genetic models) actually do not make (or need to make) that restriction (Helanterä & Uller, 2010, 2020; Day & Bonduriansky, 2011; Rivoire & Leibler, 2014; Prasad *et al*., 2015; Charlesworth, Barton & Charlesworth, 2017; Luque, 2017), and some epigenetic variants (epialleles or epiQTLs) behave virtually identical to genetic variants in terms of stable inheritance and phenotypic effects (e.g. Cortijo *et al*., 2014; Banta & Richards, 2018; Gahlaut *et al*., 2020). In fact, many estimates of supposedly additive genetic variation may well involve significant portions of non‐genetic variance (Tal, Kisdi & Jablonka, 2010; Danchin *et al*., 2011; Banta & Richards, 2018; Danchin, Pocheville & Huneman, 2019*a*). In addition, cultural evolution is already a widely used term (El Mouden *et al*., 2014; Whiten, 2019), even though it describes the change of non‐genetically inherited behavioural variation across generations (with our own species and its evolution of technologies, languages and religions as a prime example; El Mouden *et al*., 2014) and for that reason is sometimes excluded as being evolution (Charlesworth *et al*., 2017). In our opinion, the usual textbook description of evolution is a valid but restrictive special (gene‐centred) case of a more general process involving change in non‐genetically inherited traits. We think that including non‐genetically inherited traits gives us a richer and more realistic picture of evolution (see also Helanterä & Uller, 2010, 2020; Danchin *et al*., 2011, 2019*a*,*b*; Laland *et al*., 2015; Jablonka, 2017; Bonduriansky & Day, 2018; Sentis *et al*., 2018; Jablonka & Lamb, 2020). To maintain the difference and to allow for plurality in personal views, we may therefore distinguish between genetic and non‐genetic evolution (a suggestion made to us by Troy Day; see also Archer, 2015).

Moreover, adding non‐genetic inheritance gives us a broader view of natural selection, disentangling natural selection from any specific mechanism of inheritance and, therefore, recovering Darwin's original formulation of selection. This, of course, goes against some formulations of natural selection, which conceptualises it as acting only on genetic heritable traits (Endler, 1986; Michod, 1999). But, again, theoretically there is no necessity for such a limitation since natural selection will act on both genetic and non‐genetic traits.

In addition, our model shows there are additional pathways by which adaptation can be achieved by populations, apart from natural selection. In this sense, adaptive responsiveness and non‐genetic inheritance enable a second driver of adaptive evolution. While a similar pathway is not possible for genetic inheritance (since, as far as we know, the changed phenotype does not affect the genotype), non‐genetic inheritance circumvents this limitation. We discuss this second driver in more detail below.

### (3) Are organismal responses expected to be adaptive?

We argued that adaptive evolution can be driven by the non‐genetic inheritance of adaptive parental responses, but why would parental responses be adaptive in the first place? Of course they may be neutral or even maladaptive, as mentioned earlier (which would then still leave the non‐genetic inheritance of parental responses as an evolutionary process, just not one for adaptive evolution). However, many responses could concern previously evolved adaptive plasticity. Plasticity is expected to evolve to be adaptive when there is heritable variation in it and it influences reproductive success, i.e. if some plasticity outcomes are better than others then natural selection favours the better ones (Via & Lande, 1985; de Jong, 1995; Schlichting & Pigliucci, 1998; Lande, 2009). For plasticity to be able to be adaptive in the future, it is favourable that the selective environment is not a unique event but an evolutionarily recurrent situation. Because in heterogeneous environments it is often favourable to be responsive, adaptive plasticity is ubiquitous and found across all life forms.

Previously evolved adaptive responses to predictive cues is not the only reason why parental responses are expected often to be adaptive. In some cases, organisms can evaluate current feedback from different responses and implement the better response, for example by trial‐and‐error learning or by comparing the responses by other individuals (Galef & Laland, 2005; Creanza *et al*., 2017; Whiten, 2019). Similar processes involving feedback loops may also operate at other, including physiological, levels (Soen, Knafo & Elgart, 2015). Overall then, organismal responses will frequently be adaptive, or at least often enough to be biologically relevant.

### (4) A more active role for organisms in evolution

These adaptive responses provide the driving force in the second kind of adaptive evolution that we describe. Normally this driving force is provided by natural selection, i.e. differential production of *number of offspring*. But for the second type of adaptive evolution the driving force is provided by the organism itself, or what we might call differential production of *traits*. A more active role of the organism in the evolutionary process is increasingly discussed in the literature (Lewontin, 1983; Sultan, 2015; Walsh, 2015; Godfrey‐Smith, 2017; Baedke, 2020). For example, while gene flow is typically treated as an evolutionary process that randomises gene pools and therefore counters the local adaptation of populations, when individuals actively choose their environments gene flow can actually favour local adaptation (Edelaar & Bolnick, 2012; Jacob *et al*., 2017) and even result in adaptive speciation (Berner & Thibert‐Plante, 2015; Nicolaus & Edelaar, 2018). Of course similar active roles are played by individuals in the examples discussed earlier on epigenetic marks and niche construction.

This has an important consequence for how we view evolution. We often think and talk about organisms as being exposed to environmental conditions, and adapting themselves across generations. In other words, the environment is the selective agent (even when this environment is composed of other organisms, e.g. in the context of sexual selection) and the organism the selective target. But when evolution is driven by organismal responses that are passed on non‐genetically, the response of the organism provides the driving force behind adaptation and so the organism becomes the selective agent. Organismal agency has had a bad history because it has been aligned with sentient action, with mystical ‘vitalistic’ powers, or with the idea that organisms have a desire and goal to evolve to (Laland *et al*., 2019). These are not our arguments – we simply stress that organisms often exhibit changes in their traits such that they increase expected fitness, i.e. the population changes in an adaptive direction. Such directional, fitness‐enhancing change is fully expected to evolve under natural selection (Via & Lande, 1985; de Jong, 1995; Schlichting & Pigliucci, 1998; Lande, 2009), but once evolved it can combine with non‐genetic inheritance (also expected to evolve to enhance fitness: Rivoire & Leibler, 2014; English *et al*., 2015) to create a second type of adaptive evolution. While some may argue that this idea of organismal agency might arise or appeal because it reflects a more organism‐friendly (‘less cold’) version of evolution (Welch, 2017), we think it simply aligns better with reality and it is time for the organismal agency‐pendulum to swing back from its historical rejection to some balanced intermediate position.

### (5) Understanding adaptive evolution using all the terms of the Price equation

This second driver of adaptive evolution is compatible with the Price equation, since it is part of the second term (the expectation term) of the equation (Equation 1). Historically, this second term has been rather ignored by researchers due to two main, albeit related, reasons. First, the Price equation has always been considered as foremost a general description of selection (including by George Price himself), with researchers focusing mainly on the covariance term (Frank, 2012*a*; Luque, 2017; Queller, 2017). Second, natural selection has traditionally been considered the only evolutionary driver that systematically produces adaptation. In doing so, any other evolutionary driver must have, in principle, a negative or null role on adaptation (at least on average, e.g. ignoring accidental and short‐lived adaptation due to stochasticity). One possible important influence for this position was Fisher's (1930) Fundamental Theorem of Natural Selection. In its full form, the theorem mirrors the Price equation (but focusing on fitness as the focal trait) simplifying the Price equation to the sum of (1) the additive variance in fitness and (2) the expected change in fitness between generations,

(2) $$\Delta \bar{w}=\;{\mathrm{Var}}_{\mathrm{add}}\left(w\right)\;+\;E\left(w∆w\right)$$

where the second term is usually thought to be negative (i.e. not an adaptive driver). Fisher considered this theorem as a quasi‐conservation principle (Frank, 2012*b*) where the increase of mean fitness by the action of selection is counterbalanced by the second term (what Fisher called ‘environmental change’ or ‘deterioration of environment’). These two reasons may explain why the second term has historically been ignored as a contributor to adaptive evolution (see also Helantarä & Uller, 2010; Queller, 2017). Here we show that a second driver of adaptive evolution (the non‐genetic inheritance of adaptively acquired traits) is numerically independent of natural selection and is part of this neglected second term. Other potential sources of adaptation (e.g. biased mutation, biased development) are also captured by this second term, which therefore might benefit from more theoretical scrutiny (El Mouden *et al*., 2014; Helanterä & Uller, 2010, 2020).

### (6) Is there really a second driver of adaptive evolution besides natural selection?

One argument against non‐genetic inheritance of parental responses being a driver of adaptive evolution is that only natural selection can respond to novel ecological challenges (e.g. Futuyma, 2017; Bonduriansky & Day, 2018). Indeed, with respect to fully novel ecological challenges, evolution *via* parental responses is potentially limited – responses might not be adaptive in that case, which could therefore even lead to maladaptive evolution. However, many if not most ecological challenges are not novel, since environments vary across a rather constant range of spatial or temporal variation (e.g. temperature, food availability, predator density), which is exactly why adaptive responses have evolved in the first place – to be able to deal with this range of variation (Via & Lande, 1985; de Jong, 1995; Schlichting & Pigliucci, 1998; Lande, 2009). In addition, it is hard to imagine a novel ecological challenge that is not qualitatively comparable to some degree to something that has been dealt with before during the evolution of the response. For example, if a non‐native predator using unfamiliar attack mechanisms and strategies is introduced somewhere, some general prey responses such as hiding, living in groups, or developing a different size might still reduce the probability of being predated. Alternatively, quantitatively novel challenges (e.g. an unprecedented heat wave) may still be somewhat addressed by the extrapolation of an organism's response to evolutionarily familiar challenges (normal variation in temperature). These general or extrapolated responses are probably more likely than not to be adaptive, even if the challenge is novel.

Another argument is that parental responses do not lead to an accumulation of adaptation over many generations, as is the case for genetic adaptation *via* natural selection. Step by step, genetic adaptation *via* natural selection can result in remarkable and clearly adaptive traits like an eye. Indeed, genetic evolution is incremental and builds on previous genetic changes, and is open‐ended – future eyes may be even more remarkable, or evolve into something with a different function. Parental responses are generally much more restricted as they represent some response out of a limited set of potential responses (e.g. the range of phenotypes that can be produced by a reaction norm). While this is certainly true, we do not think that open‐endedness is a defining character of adaptive evolution, especially since genetic evolution also has certain limits and constraints so is not open‐ended in all respects (there are physical, genetic and developmental constraints imposed on evolution; Rasskin‐Gutman, 2005; Gavrilets, 2010). In addition, some parental responses could also result in open‐ended cumulative evolution, for example non‐genetically transmitted behaviours or vocalisations.

Yet another argument supporting that only natural selection can drive adaptive evolution is that the parental response for a trait could only have evolved to be adaptive because of prior natural selection, and the same can be said about many mechanisms of non‐genetic inheritance (e.g. Dickins & Rahman, 2012; El Mouden *et al*., 2014; Rivoire & Leibler, 2014; Futuyma, 2017). We fully agree with this. But the question then is at what level of causality we want to provide an explanation for the observed (or desired) match between organism and environment. When looking at life as a whole, the differential reproductive success of the evolving entities and therefore natural selection is the more ultimate explanation. However, when we are concerned with more short‐term bouts of adaptation (and evolution is often seen and defined as occurring between two consecutive generations), then natural selection may play a more modest role, and the evolution of acquired traits might be present and even have a numerically important role. Hence, especially for ongoing evolution and practical applications (crop improvement, pest control, management of endangered species, etc.), this second type of adaptive evolution might be very relevant and should therefore not be ignored.

### (7) Evolution involves a gradient of temporal stability of what evolves

That non‐genetic evolution might play a role especially in adaptation at smaller temporal scales is in part because of the stability of the transmitted traits. At the extreme, some parental traits are only transmitted for one generation (sometimes called intergenerational effects), whereas others can be transmitted for two (multigenerational effects) or more than two generations (transgenerational effects; Wang *et al*., 2017). DNA is sometimes given the status of immortality. However, this obviously cannot be the case if genetic evolution is to happen in the long term – also DNA sequences mutate and are therefore somewhat unstable. Hence, the lower stability of non‐genetic variants compared to genetic variants is a matter of degree (stability also varies between genetic variants, e.g. microsatellites have much higher mutation rates than other sequences), not a distinguishing feature. Note however that the decay or reversion of non‐genetic traits does reduce our ability to detect past non‐genetic evolutionary events: they may simply not leave the type of detectable trace that DNA does record (unless an epigenetic change is converted into a genetic change; Danchin *et al*., 2019*b*).

The lower stability of some non‐genetic traits is not necessarily an evolutionary weakness – it might be their strength. Environmental change occurs over different temporal scales. For example, climate change occurs over cycles spanning tens of thousands of years (at least, until recently), whereas other environmental variables may cycle over just a few years (e.g. El Niño cycles, predator–prey cycles, etc.). Genetic adaptation tends to be slow: even under moderately strong natural selection, alleles take many generations to reach fixation (especially in large populations, as the approach to fixation is asymptotic). When environmental change is slow relative to generation time, genetic evolution can keep up with environmental change. But when environmental change is relatively fast, genetic evolution lags. It is here that the transmission of parental responses might provide a much faster evolutionary change. At the extreme, if all individuals of the parental generation respond adaptively to some newly present ecological challenge or opportunity and transmit their response to the next generation, then the ‘fixation’ of the adaptive trait only takes a single generation (compared to many generations in the case of genetic evolution). Once this ecological challenge has disappeared, the population should return rapidly to its initial state, and here a high conversion or decay rate allows exactly that. For ecological challenges that occur over somewhat longer temporal scales, a lower decay rate might be more advantageous (especially if environmental stochasticity between generations is involved, so that populations do not respond to these erratic changes but to the underlying trend). It has therefore been hypothesised that the various means of transmission (genetic and various non‐genetic ones) have evolved to match the temporal scales of different ecological challenges, to allow for an adaptive diversification in how and how rapidly an evolutionary response is brought about (Jablonka *et al*., 1995; Helanterä & Uller, 2010; Rivoire & Leibler, 2014; Kuijper & Hoyle, 2015; English *et al*., 2015; McNamara *et al*., 2016; Walsh *et al*., 2016; Bell & Hellman, 2019; Danchin *et al*., 2019*a*; Anastasiadi *et al*., 2021). So also here it may be beneficial to recognise not a binary division but a gradient in evolutionary stability of traits (including within the genetically inherited traits, e.g. some highly conserved genes have much lower mutation rates than microsatellites; see also Monroe *et al*., 2022), and to consider that evolution might be more rapid and dynamic than typically assumed.

Overall then, we certainly do not want to diminish the general importance of natural selection nor that of genetic inheritance for adaptive evolution. However, we do believe that in many specific cases (including those of practical relevance) adaptive evolution *via* the inheritance of acquired traits has played or can play a significant role. Only the evaluation of this possibility (and our causal model can help with this) will tell us to what extent this is true.

### (8) Limitations to our approach

What are the limitations to the causal model we presented, and is it general and flexible enough? Our causal model captures all forms of adaptation as recognised in Edelaar & Bolnick (2019). It does not necessarily differentiate between them, e.g. the same causal connection captures whether an environment is adjusted or chosen. Nevertheless, all forms of achieving organism–environment match can be represented by choosing the appropriate variables (including those of higher orders, like a variance or a density) and causal connections, so in that sense it appears it cannot be more general. It also simplistically but comprehensively includes all forms of inheritance, in the sense that all inheritance is by definition genetic or non‐genetic. With respect to the traits that can evolve, we focussed on classical and extended phenotypes, and extended the concept of the extended phenotype even more by including that chosen environments are also part of the extended phenotype. Again based on Edelaar & Bolnick (2019), this appears to cover all options, since any relevant extended phenotype can be captured by defining the variables of the causal model as necessary, e.g. the sexual partner or the symbiont. If additional aspects of the extended phenotype are recognised or uncovered, we expect these can simply be incorporated as one of the focal traits.

While in those senses very general and flexible, our model does have limitations. Of course it is very simplified and abstracted. A complex process like the development of an organism from genetic and other internal and external (environmental) inputs is captured by just a few variables and causal connections. The genetic input itself is also very simplistically described, and leaves out real biology like polygenic causation, epistasis, dominance, pleiotropy, modularity, etc. We did not include this additional detail because we wanted to show the heuristic value of the approach, and the insight that is already gained from it. These extra details can be added to the model by the user if desired, to see whether this provides additional insight or whether the simplification is good enough (as in quantitative genetic models, which also simplify much yet have proven very useful, both empirically and theoretically).

Another important simplification we made is the assumption of linearity, which is reflected in our focus on the first‐order Price equation (thus ignoring higher‐order moments) and the assumption that different causal pathways are ‘added up’ to yield the total evolutionary change. When effects are non‐linear functions of their causes, these assumptions do not hold, or at best outcomes of the model are first‐order approximations. To account for non‐linearity one needs to consider derivatives or introduce extra variables representing higher moments (Henshaw *et al*., 2020; Araya‐Ajoy *et al*., 2020). These higher moments are also captured by the Price equation (Rice, 2004).

A more challenging task is to incorporate temporal dynamics, and especially feedback loops, which are ubiquitous in development. Although these mechanisms can in principle be modelled by introducing time‐indexed variables, it inflates the numbers of parameters and thereby reduces the empirical identifiability and interpretability of the model. This might pose a limit on the application of causal models to some types of niche construction where organismal traits and environmental factors form a feedback loop or ‘reciprocal causation’ (Laland *et al*., 2011). So overall, although causal modelling is flexible and the model can be adapted at will, there is a trade‐off between precision and tractability, and a modeller needs to find an appropriate level of complexity depending on the biological system or the study question.

### (9) Viewing the causal structure as the evolving unit

The causal modelling perspective also brings a conceptual implication as to the unit of evolution, or what are those things that are said to evolve. As discussed above, the traditional view takes genes (or gene‐like entities like epigenes) as primary and ultimate actors of evolution, and conceptualises adaptive evolution as a change of genetic frequencies caused by genetic effects on fitness. Genes take primary responsibility for all that happens in development and evolution, whereas phenotypes and environment are conceived as their effects (as in ‘extended phenotype’) or background parameters. Our approach suggests a different picture, according to which evolution is an outcome of the causal nexus connecting genes, phenotypes, and environmental variables. Which among these factors takes the lead depends on how they are causally configured. In particular, when non‐genetic inheritance is present, genes are not even the ‘ultimate’ cause of development, parent–offspring resemblance, or adaptive evolution. This does not mean that genes are not major components of adaptive evolution. They are. But it does suggest that not everything can always be reduced to genetic evolution. Rather it may be the whole causal structure, consisting not just of genetic but also of phenotypic as well as environmental variables, that evolves (Otsuka, 2019*a*). For example, local adaptation across a metapopulation is not the local change in genetic composition, but the joint evolution of genotypes, the environments with which these interact, and the phenotypes that mediate this interaction. Dawkins (1982) characterised his gene‐centric (*versus* organism‐centric) view as a particular way of looking at animals and plants, just like one way of looking at a Necker cube (a three‐dimensional drawing of a cube that can be seen to face in different directions). A recent review reaches the same conclusion: the gene's‐eye view is one, but not the only, valid way to study and understand evolution (Ågren, 2021). Taking an entire causal structure, rather than the set of genes (or what Dawkins calls ‘active germ‐line replicators’), as an evolutionary unit is yet another way of looking at evolution. Neither of the different perceptions is more correct or true (*cf*. Dawkins, 1982). All are, but each sheds light on evolutionary phenomena from a different and complementary angle. Our model and its implications discussed herein are such a view from the other side, and reveal aspects of evolution which are not conspicuous from the traditional standpoint.

## VI. CONCLUSIONS

(1) Certain biological phenomena and various authors suggest that a revision of evolutionary theory would be useful. The use of causal modelling allowed us to produce a general framework for the study and understanding of adaptive evolution that can address some of the challenges. To make the framework general, incorporation of three aspects was deemed critical: (*i*) a broadened view of extended phenotypes; (*ii*) that traits can respond to each other; and (*iii*) that inheritance can be non‐genetic.
(2) Current mainstream usage of theory on adaptive evolution can be seen as specific applications of our more general framework, and we see our framework as a natural extension of older theory.
(3) The extended, more general model of adaptive evolution identifies two complementary yet independently operating drivers of adaptive evolution: natural selection on genetically and non‐genetically transmitted traits, and non‐genetic transmission of parent's adaptive responses.
(4) Adaptive evolution *via* inheritance of parental responses is captured by an often‐neglected component of the Price equation, which therefore warrants more future attention.
(5) Our causal model can serve as a heuristic tool to clarify conceptual issues (e.g. when niche construction is an evolutionary process, and when it is not) and to help design empirical research. While it is general and simplified, it can be adjusted flexibly in terms of variables and causal connections, depending on the research question and/or biological system of interest.
